# Natural Organic Matter Removal and Fouling in a Low Pressure Hybrid Membrane Systems

**DOI:** 10.1155/2014/893203

**Published:** 2014-01-08

**Authors:** Vedat Uyak, Muge Akdagli, Mehmet Cakmakci, Ismail Koyuncu

**Affiliations:** ^1^Department of Environmental Engineering, Faculty of Engineering, Pamukkale University, Kinikli, 20020 Denizli, Turkey; ^2^Department of Environmental Engineering, Faculty of Civil, Istanbul Technical University, Maslak, 34469 Istanbul, Turkey; ^3^Department of Environmental Engineering, Faculty of Civil, Yildiz Technical University, Esenler, 34220 Istanbul, Turkey

## Abstract

The objective of this study was to investigate powdered activated carbon (PAC) contribution to natural organic matter (NOM) removal by a submerged MF and UF hybrid systems. It was found that filtration of surface waters by a bare MF and UF membranes removed negligible TOC; by contrast, significant amounts of TOC were removed when daily added PAC particles were predeposited on the membrane surfaces. These results support the assumption that the membranes surface properties and PAC layer structure might have considerably influential factor on NOM removal. Moreover, it was concluded that the dominant removal mechanism of hybrid membrane system is adsorption of NOM within PAC layer rather than size exclusion of NOM by both of membrane pores. Transmembrane pressure (TMP) increases with PAC membrane systems support the view that PAC adsorption pretreatment will not prevent the development of membrane pressure; on the contrary, PAC particles themselves caused membrane fouling by blocking the entrance of pores of MF and UF membranes. Although all three source waters have similar HPI content, it appears that the PAC interaction with the entrance of membrane pores was responsible for offsetting the NOM fractional effects on membrane fouling for these source waters.

## 1. Introduction

Natural organic matter (NOM) is composed of a heterogeneous mixture of humic substances, carboxylic acids, proteins, amino acids, hydrocarbons, and polysaccharides [1–3]. Because of the complex nature of NOM, surrogate parameters of total organic carbon (TOC), ultraviolet absorbance at 254 nm (UV_254_), and specific UV_254_ (SUVA_254_) are often used to represent its general properties. The physical and chemical nature of NOM varies according to the water source, age, and season [4]. Therefore, effective removal of NOM has been a challenge for water utilities. On the other hand, chlorine can react with NOM to form disinfection by products (DBPs), which are considered carcinogenic and mutagenic [5].

Because of more stringent drinking water quality regulations, pressure driven membrane processes, such as microfiltration (MF) and ultrafiltration (UF), are increasingly popular in drinking water treatment, since conventional treatment processes, including coagulation, sedimentation, and sand filtration, may not meet the criteria. The extensive use of membranes, however, is still limited, mainly due to membrane fouling problems. Previous studies regarding the membrane filtration of surface waters have identified NOM as one of the major foulants in the membrane process [6–8]. Effective removal of NOM by MF and UF process may not be sufficient when they are solely used. To improve the membrane performance level, various hybrid membrane systems have been developed, such as coagulation-UF/MF, powdered activated carbon-UF (PAC-UF), and iron oxide adsorption-UF [8–14].

PAC-UF systems were found to be effective in the removal of organic compounds having both low and high molecular weights. It was reported that approximately 90% of humic acids was removed with operation of an initial concentration of 10 mg/L at a PAC dosage of 100 mg/L [13]. The primary role of PAC particles added to the UF system was to remove low molecular weights of hydrophilic precursor, which cannot be removed by UF alone. It was found that the addition of PAC into feed water containing humic acids caused a decrease in permeate flux with respect to membrane fouling, even though the organic removal by PAC was enhanced during UF [14–17].

The membrane-adsorption filtration systems are regarded as an alternative way to achieve a high removal efficiency of NOM in a cost-effective manner [13]. The cross-flow microfiltration hybrid system demands higher energy for the operation. However, the submerged membrane hybrid system requires only a low suction pressure, thus requiring lower energy for its operation. In this hybrid system, the entire treatment activity can be carried out in a single unit. In this system, TOC which normally can pass through the MF are pre-adsorbed onto PAC particles. The PAC together with adsorbed organics is then separated by the membrane filtration process. Literature studies showed that the addition of PAC could: (i) provide better physical removal of NOM, (ii) reduce the direct loading of dissolved organic pollutants onto the membrane, and (iii) prevent membrane fouling [18–20]. Kim et al. [21] found that the system could consistently remove more than 95% TOC with a PAC dose of 40 g/L for 40 days from a synthetic wastewater. The aim of this study was to assess the PAC pretreatment method for reducing NOM fouling of pilot scale MF and UF hybrid membranes. The main focus proposed is to assess the relative effects of PAC adsorption.

## 2. Experimental Section 

### 2.1. Source Waters Quality

Water quality is an important factor in understanding membrane fouling. Three surface waters used for studying NOM fouling with the two different low pressure membranes were Terkos, Omerli, and Buyukcekmece lake water. These surface water supplies are the major drinking water sources of Istanbul City.  Table 1 summarizes the physicochemical characteristics of these source waters. The highest SUVA_254_ value (2.81 L/mg∗m) and bromide (Br^−^) ion concentration (230 *μ*g/L) were observed in Buyukcekmece water, while a low to moderate level of TOC and UV_254_ value of 4.52 mg/L and 0.100 cm^−1^, respectively, was measured in Omerli water.

### 2.2. Membranes and PAC

**Figure 1:**
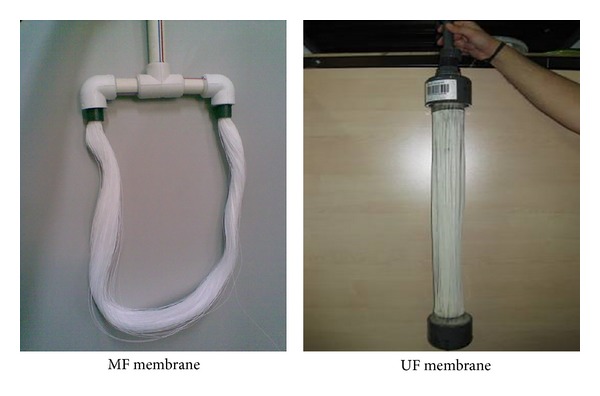
A general photographic image of MF and UF membranes.

The NOM rejection and membrane fouling were performed on commercial polypropylene hollow fiber MF (Zena Membranes, Czechoslovakia) and ZW-10 model UF membranes (Zenon Environmental Inc., Canada) (Figure 1). The module operated at constant flow in an outside/in type of configuration. The operating vacuum pressure provided by the pump induces a flow of water from outside to the inside of the membrane fibers. Each membrane was anticipated to show different trends of membrane fouling depending on source waters characteristics and membrane properties. Both of MF and UF membranes have surface area of 0.93 m^2^, respectively. The pore sizes of MF and UF membranes are 0.10 and 0.047 *μ*m, respectively. Besides, Table 2 lists the relevant properties of these membranes according to their manufacturer and data available in the literature. On the other hand, AquaSorb BP2 PAC was used for adsorption purposes. AquaSorb BP2 PAC has 27 *μ*m average pore size, 1.56 cm^3^/g pore volume, and 950 m^2^/g surface area. The PAC was firstly mixed with water in a beaker, and then its solution was added into the first part of reactor.

### 2.3. Experimental Hybrid Membrane Filtration Setup

**Figure 2:**
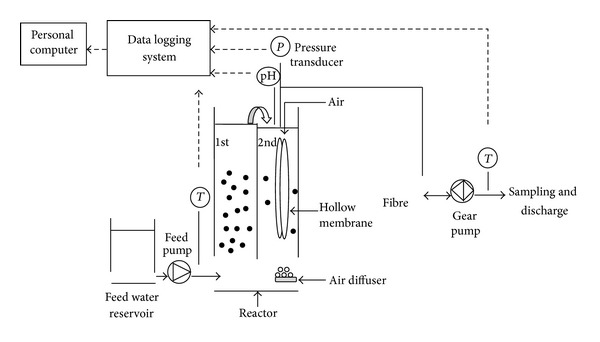
A schematic diagram of pilot plant.

MF and UF experiments were performed in a submerged hybrid membrane system (Figure 2). Prior to experiment, all membranes were cleaned with deionized (DI) water and compacted at operating conditions. As seen in Figure 2, surface lake waters were taken from the 1,500 liter tank by peristaltic pump and transferred into the membrane reactor. The hybrid submerged membrane reactor is made of Plexiglas. The volume of the reactor was 30 liter and it was separated into two parts with a baffle system. The first compartment serves as an adsorption zone, while the second part was used for submerged membrane filtration. The raw water was transferred into first part of the reactor which the water was firstly contacted with PAC and then passed into the second part containing submerged membrane by a bottom canal. Water level sensor was located at the first part of the reactor to be kept constant water level in the reactor. Permeating and backwashing operations were performed automatically with automatic control system. Pressure gauge was placed in the vacuum line in order to measure transmembrane pressure (TMP). All measured data were monitored online and stored by HACH model SC1000 data logging system. Samples were taken once a day from the permeate line, reactor, and the raw water tank. Permeate flow rate was kept constant and was monitored daily during the experiments. The operating fluxes for MF and UF systems were set as 150 L/m^2^-h and 18 L/m^2^-h, respectively. Air was supplied from a porous ceramic plate below the membrane module in order to provide dissolved oxygen to create turbulence along the membrane surface which helps to remove particles that deposit on the outside of the membrane fiber. Experiments continued for one week and both MF and UF membranes were cleaned between each experiment. Different chemicals were used during chemical cleaning procedure of each membrane. These two membrane modules were cleaned according to the following steps: (a) surface cleaning with DI water, (b) acidic wash in 2% HCl solution for 2 hours, (c) basic wash with 1 N NaOH solution for overnight, and (d) final cleaning in 0.4% NaOCl for 2 h. Membranes were rinsed with DI water after every step and prior to all experiments [21].

### 2.4. Analytical Methods

**Figure 3:**
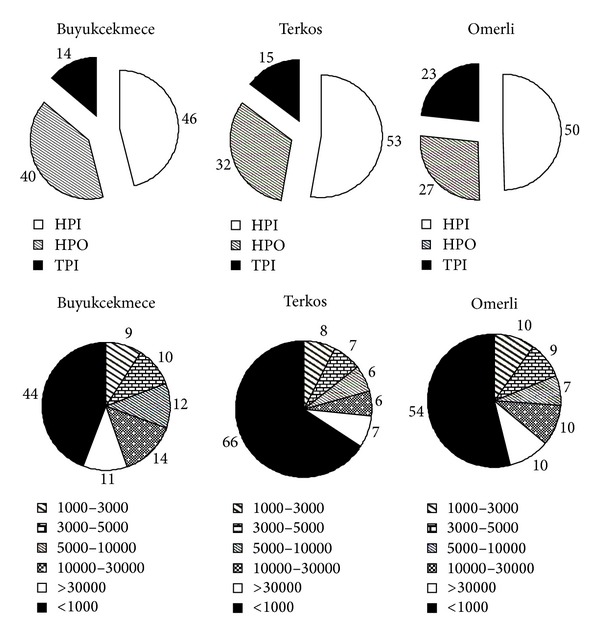
Chemical NOM fractionation and molecular weight distribution of source waters (%).

TOC analysis was employed by high temperature combustion according to Standard Methods (SM) 5310 B using a Shimadzu TOC-VCPH analyzer equipped with an auto-sampler [23]. The instrument provided reliable, accurate, and reproducible data with a minimum detection limit of 2 *μ*g/L C. Further, UV absorbance measurements were determined with a Perkin Elmer Lambda 25 UV Visible spectrophotometer at a wavelength of 254 nm. On the other hand, a Superlite DAX-8 (Supelco, USA) and Amberlite XAD-4 (Rohm and Haas, Germany) was used to fractionate NOM into three groups, that is, hydrophobic (DAX-8 adsorbable), transphilic (XAD-4 adsorbable) and hydrophilic (neither DAX-8 nor XAD-4 adsorbable) fractions. The chemical fraction of NOM contained source waters was displayed in Figure 3. The molecular weight distribution of the source waters was determined using sequential filtration through membranes of decreasing molecular weight cut off (MWCO) [24]. Source waters were fractionated in a 76 mm diameter stirred cell (model 8400, Amicon, Beverly, MA, USA) using a serious of regenerated cellulose acetate UF membranes from Millipore designated YM30, YM10, YM5, YM3 and YM1 with nominal MWCOs of 30, 10, 5, 3 and 1 kDa, respectively. The distribution of molecular weights is shown in Figure 3. Other parameters such as turbidity, pH and conductivity were also determined with online monitoring system (Hach-Lange SC1000).

## 3. Results and Discussion

### 3.1. NOM Reduction by PAC Pretreatment

**Figure 4:**
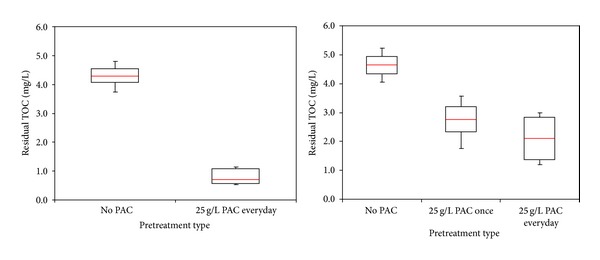
TOC removal with MF and UF in Terkos water.

**Figure 5:**
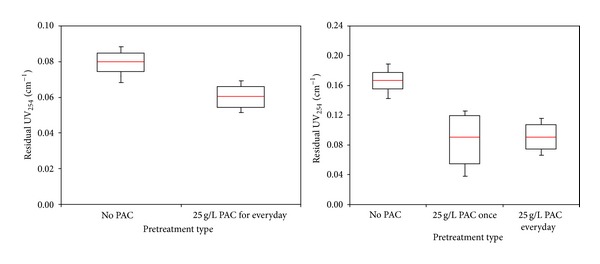
UV_254_ removal with MF and UF in Terkos water.

**Figure 6:**
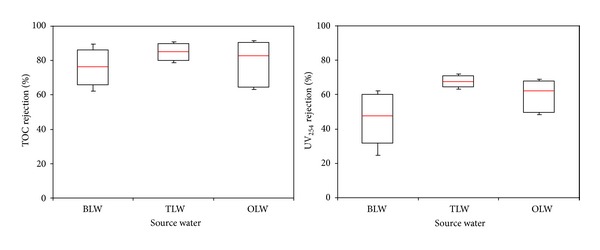
TOC and UV_254_ removal with PAC/MF for Istanbul water sources.

**Figure 7:**
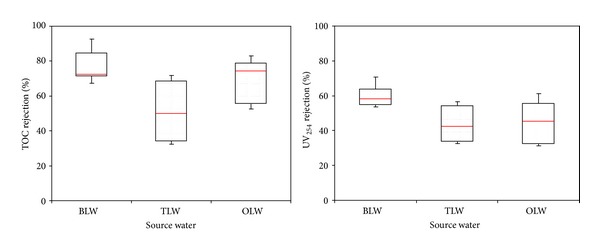
TOC and UV_254_ removal with PAC/UF for Istanbul water sources.

Removal of organic precursor by membrane process is impacted by many factors such as water chemistry (pH, ionic environment), NOM characteristics (hydrophilic-hydrophobic character, MW distribution), and membrane properties (pore size, hydrophilicity, surface charge) [8, 25–27]. The performances of the MF and UF membrane-adsorption hybrid systems in removing NOM compounds, with respect to TOC and UV_254_ rejection for the 3 water samples analyzed, are summarized in Figures 4–7. The efficiency of the membrane-adsorption hybrid system depends on PAC addition mode and raw water properties [19]. The effects of PAC addition to MF and UF membrane filtration performances were examined with a pilot submerged hybrid membrane system. To learn the effects of the presence of PAC concentration on membranes performance, addition of PAC into MF and UF membrane reactor was performed with different modes. These different PAC methods of UF are classified as No PAC addition, 25 g/L PAC addition once during the study period, and 25 g/L PAC addition every day. On the other hand, a PAC addition mode of MF membrane reactor was defined as No PAC addition, and 25 g/L PAC addition every day. During the pilot plant studies, samples collection from feed and permeate line were conducted once a day for seven days period. The results were evaluated with residual TOC and UV_254_ value of permeated water.

The box plots of the comparison of residual TOC and UV_254_ level of adsorbent/membrane systems with different PAC methods for Terkos water are provided in Figures 4 and 5. The median value of organic precursors was demonstrated with red line located inside of each box. Moreover, the median residual TOC level for two PAC conditions with MF membrane was found to be 4.45 and 0.76 mg/L, respectively (Figure 4) while, PAC/UF hybrid system lowered the median residual TOC concentration for three PAC conditions to 4.61, 2.83, and 2.19 mg/L, respectively (Figure 5). Filtration of Terkos water by a bare MF and UF membranes removed negligible TOC (<18% for MF and 13% for UF); by contrast, significant amounts of TOC (85% for MF and 51% for UF) were removed when 25 g/L-day PAC were predeposited on the both of membrane surfaces (Figure 4). Since the MF membrane pore diameter (0.1 *μ*m) is too large compared to molecular size of NOM present in three source waters (Table 1), the dominant removal mechanism of PAC/MF hybrid system is adsorption of organic matter within PAC layer rather than size exclusion/steric hindrance of NOM by membrane pores. On the other hand, raw waters background ionic strength (Ca^+2^ concentrations) attenuates potential electrostatic repulsions between NOM molecules and the membrane. As indicated by Ates et al. [28]; the bare MF membrane rejects relatively large particulate molecules more, showing that the colloidal and dissolved NOM compounds are preferentially transmitted through the membrane pores, as its molecular size is relatively small. In other words, organic substances can easily pass through MF membrane without PAC treatment. However, in the case of PAC/MF hybrid system, the PAC particles was adsorbed onto the surface of membrane pores and prevent the transition of NOM molecules and this resulted in higher level of organic precursor removals compared to the bare MF membrane. Thus, the accumulation of high and low molecular weight organics inside the PAC layer was prompted, while the NOM fouling mode was transformed from pore plugging to cake formation at the entrance of MF pores.

On the other hand, the organic precursors of TOC and UV_254_ removals efficiency of UF membrane were found to be less than MF membrane in the presence of PAC (Figures 4 and 5). These results support the assumption that the surface related properties (surface roughness and surface charge) of membranes and PAC layer structure (surface deposition) might have considerably influential factor on organic precursors removal. In the case of 25 g/L PAC addition for every day, the average TOC removal efficiency of PAC/UF system (Terkos water) was found to be 51% (Figure 4). As can be seen from Figure 5, lower average UV_254_ rejection (68% for MF and 44% for UF) was observed with PAC/MF and PAC/UF systems. Inconsistent with the literature findings, the PAC removed UV_254_ less efficiently than TOC, suggesting that they selectively bind more hydrophilic molecules. Furthermore, these findings indicate that PAC adsorbent preferentially removes aliphatic organics to which UV_254_ is not attributed to [29]. With PAC/MF hybrid system for three Istanbul surface waters, average TOC removals were at or above 80%, while average UV_254_ rejections were at or under 59% indicating different rate of organic precursor removal efficiencies (Figure 6). It was also reasonable to expect that the adsorption of hydrophilic compounds, which are enriched in proteins [30], occurred at the organic amino groups and carboxylic sites of PAC. These findings are in agreement with Henderson et al. [31] and Campinas and Rosa [19]. Moreover, as summarized in Figure 6, the highest and lowest average TOC (85% versus 76%) and UV_254_ (48% versus 68%) removal were observed for Terkos and Buyukcekmece waters, respectively. Furthermore, it is clear from Figure 6 that the overall PAC/MF hybrid system rejection efficiency of TOC and UV_254_ remained in the middle range for Omerli water.

The data of UF system demonstrate that UF alone reject negligible TOC and UV_254_ (Figure 5) as found by Li and Chen [17]. On the other hand, Figure 7 depicted that during the PAC/UF operation, the highest average TOC removal efficiency (76%) was observed for Buyukcekmece water, while the lowest average TOC removal efficiency of Terkos water was found to be 50%. Interestingly, even though Terkos water has the highest TOC and UV_254_ removal with PAC/MF system, the PAC/UF hybrid system resulted in lowest TOC and UV_254_ rejection for same water samples as summarized in Figure 7. These findings indicate that the PAC particles accumulated onto the MF membrane pores at short time and this resulted in more NOM related compounds removal. In fact, most of the rejection of organic precursor occurred inside the PAC layer not at MF membrane surface. On the other hand, NOM structure and organic and inorganic properties of raw water have a significant impact on organic precursor rejection with two different membranes. As summarized in Figure 7, the NOM was removed more efficiently by the PAC/MF system than PAC/UF system. Furthermore, lower organic precursor rejections by PAC/UF hybrid membrane process could be essentially related to the exclusion of PAC particles (27 *μ*m) by UF membrane pores (0.04 *μ*m). It was concluded that the UF membranes reject PAC particles more, showing that the low molecular weight (<1 kDa) organics (Terkos; 66%, Buyukcekmece; 44%, and Omerli; 54%) are preferentially transmitted through the UF membrane pores, as its molecular size is relatively small compared with that of its aromatic counterpart in raw water samples. Furthermore, the enhancement of organic precursor removals with PAC/MF system is probably explained by the pore blocage caused by PAC adsorption to the porous surface and/or by the additional sieving provided by the PAC deposits onto the MF membrane surface, as observed by others [19, 24].

It is important to note that the presence of dissolved Mg^+2^ and Ca^+2^ in the waters of Buyukcekmece, Terkos, and Omerli, has a strong influence on the UF membrane performance. The Ca^+2^ ions can bind with the acidic functional groups of the NOM, elevating the degree of hydrophobicity of the NOM molecules and developing a dense thick fouling layer on the UF membrane surface [32]. The findings of Lohwacharin et al. [16] indicated that the addition of Ca^+2^ into River Obitsu significantly improved the NOM rejection in the presence of carbon black (CB), and they reported that the increased removal of NOM by addition of Ca^+2^ was caused by the intermolecular bridging of hydrophilic compounds and CB induced by Ca^+2^, notably at pH 7.7. As can be seen in Figure 7, the removal efficiency of TOC and UV_254_ precursor compounds shows an increase with Ca^+2^ concentration of source waters (Table 1). However, the MF performance of water with higher Ca^+2^ content was largely or even mostly influenced by the PAC particles accumulation onto the MF membrane pores, rather than by the Ca^+2^ complexation with NOM compounds in source waters.

### 3.2. NOM Fouling Potential of Hybrid Membrane System

**Figure 8:**
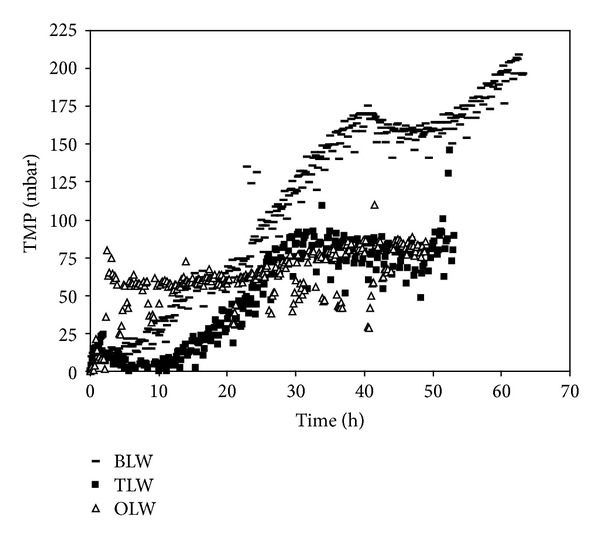
TransMembrane pressure versus time for PAC/MF system.

**Figure 9:**
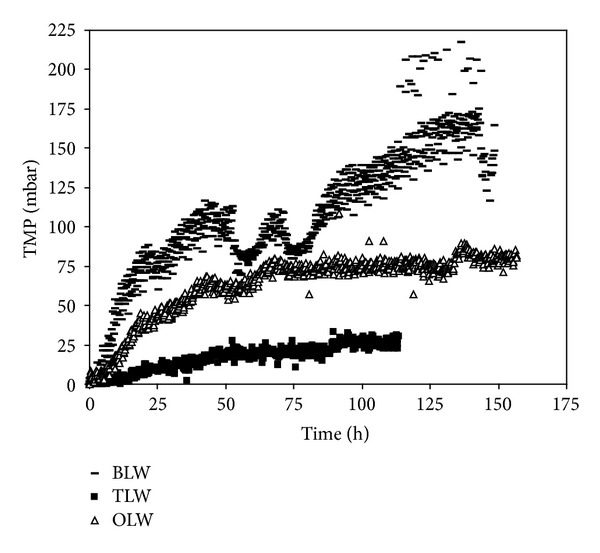
TransMembrane pressure versus time for PAC/UF system.

This section focused systematically on the effects of membrane type, source waters chemistry, and PAC pretreatment on membrane fouling. In order to compare fouling data obtained with three surface waters containing different concentration of TOC, MF, and UF, membrane fouling profiles are plotted as a function of filtration period (Figures 8 and 9).  Figure 8 shows the variation of MF membrane fouling obtained with different sources of NOM. Based on filtration period, Buyukcekmece water resulted in the greatest membrane fouling, while Terkos water and Omerli water followed similar fouling trends after 30 h filtration time. The maximum TMP values of source waters were found to be 100, 200, and 90 mbar for Terkos, Buyukcekmece, and Omerli waters, respectively. Considering the dominant chemical NOM fraction of these source waters (Figure 3), these data suggest that all three surface waters should have had similar fouling trend. However, Buyukcekmece water which has a hydrophilic (HPI) fraction of 46% resulted in the highest fouling potential compared to Terkos and Omerli waters. Furthermore, two source waters of Terkos and Omerli water showed a similar fouling pattern since they have similar HPI fraction percent as shown in Figure 3 (53% versus 50%). It was concluded that since all three source waters have similar HPI content (Figure 3), it appears that the PAC interaction with membrane pores was responsible for offsetting the NOM fractional effects on membrane fouling for all three source waters. Besides, Zhao et al. [18] concluded that hydrophobic/hydrophilic character or the molecular size distribution ratio of NOM plays a quite limited role on PAC cake formation, while a more relevant effect is due to the combination of ionic strength, colloidal movement, PAC pore constriction, and pore blockage mechanisms. Several studies indicated that the fouling caused by gel layer formation and concentration polarization is less severe that caused by the pore adsorption and pore blocking mechanisms [33–35]. It was reported that both pore adsorption and pore blocking mechanisms directly block the narrow passage, that is, the membrane pore, for water, while gel and concentration polarization layers forming on top of membrane surface do not directly block the passage of water. Therefore, the extent of membrane fouling by these mechanisms will be quite different [17].

Solution chemistry has an important effect on the surface charge characteristics of polymeric membranes. Moreover, the higher level of conductivity (540 *μ*S/cm) and TDS (258 mg/L) parameters (ionic composition) combining effects (Table 1) in Buyukcekmece water is supposed to be responsible for higher NOM fouling compared to other two source waters. As reported in several literature studies, especially the intensity of high ionic strength of this source water prompted the membrane fouling further. It is generally agreed that NOM fouling is more severe in a high ionic environment [24, 36]. The reason for the deleterious effect of solution high ionic strength is that at high ionic strength NOM molecules are smaller and their configuration are more spherical leading to augmented diffusivities. This promotes significant diffusion of NOM molecules into the membrane pores, leading to enhanced membrane fouling due to pore adsorption as was the case with Buyukcekmece water. High ionic strength may also compress the double layer of the NOM molecules, leading to intensified aggregation and cake formation [37]. As a result, the cake formed in a high ionic strength solution is more compact and has higher resistance [38]. Therefore, the membrane fouling increases faster as was the case with Buyukcekmece water at high ionic strength than at those of low ionic strength, such as Terkos and Omerli waters.

In our results, adsorption of NOM onto the MF and UF membrane increased with increased level of Ca^+2^ for Buyukcekmece water (Figures 8 and 9), probably due to reduction of electrostatic repulsion. This is consistent with other studies reporting increased adsorption by electrostatic charge shielding, complexation, and/or bridging effects [39]. Our results also indicate that Ca^+2^ induced a change in the NOM fouling mechanism, which was associated with remarkably more NOM deposited on the membrane. It is believed that Ca^+2^ played a crucial role in the formation of lower level NOM fouling with Terkos and Omerli waters. We suppose that higher level of NOM fouling with Buyukcekmece water (Ca^+2^: 161 mg/L) is caused by calcium complexation, which is reported in literature and known as the egg-box model [40]. This gel probably caused the increase in membrane fouling because it is tighter and less permeable than the cake layer formed with Terkos (Ca^+2^: 46 mg/L) and Omerli (Ca^+2^: 30 mg/L) waters. Furthermore, as the pH increases, the membrane surface and pores become more negatively charged due to the presence of anions. As a result, the pore size of the membrane is reduced because of the repulsion between neighbor negatively charged groups and adopts an extended conformation. As it was stated by many studies, alkaline pH showed higher NOM rejections and membrane fouling.

On the other hand, Figure 9 shows the variation of UF membrane fouling obtained with different source waters contained NOM. PAC is expected to compete with the UF membrane for the adsorption of NOM compounds that otherwise would adsorb on the membrane, causing its fouling. However, some authors have indicated that although PAC itself does not impose significant membrane fouling, when in the presence of NOM it increases the fouling resistance [17, 41]. It is believed that NOM acts as a glue that binds the PAC particles to one another and to the membrane surface, enhancing fouling. For direct comparison purposes, TMP development curves for both hybrid systems are depicted in Figures 8 and 9, respectively. It was found that Buyukcekmece water resulted in the highest NOM fouling as compared to other two water sources. While the TMP value of 200 mbar was obtained for 65 h with PAC/MF system, in the case of PAC/UF hybrid system, the 200 mbar value of TMP was occurred after 150 h filtration period. This findings revealed that NOM fouling of PAC/UF membrane developed for longer filtration times (>150 h) than those of MF membrane (<70 h). Since PAC particles were not be able to diffuse the UF membrane pores effectively, the membrane fouling periods of UF membrane resulted in longer than those of MF membrane. It was assumed that the structure of surface cake layer of UF membrane was more permeable than those of MF module; thus, the NOM fouling period and intensity of MF took place earlier than those of UF system as shown in Figures 8 and 9.

**Figure 10:**
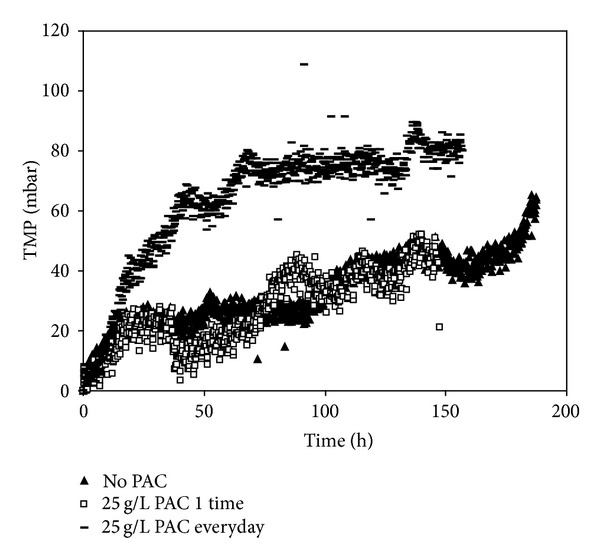
Comparison of transmembrane pressure values in terms of PAC addition modes in PAC/UF system of Omerli water.

In order to investigate the effects of PAC addition modes on UF TMP development, three different PAC addition methods were performed with UF system for Omerli water.  Figure 10 shows significant differences at all PAC addition methods, which indicates that 25 g/L PAC addition for every day promoted the membrane fouling more. This same trend has already been obtained by other authors [8, 14, 42]. Moreover, no substantial differences were obtained for No PAC and 25 g/L PAC 1 time addition for Omerli water. This result indicates that the amount of PAC added into UF membrane system plays a crucial role on TMP development. In other words, accumulation of PAC particles on the surface of UF membrane accelerates the higher level of NOM fouling. These findings also indicate that the higher level of organic precursors rejection with MF membrane is attributed to less permeable cake layer of MF compared to more permeable cake layer of UF module.

It is clear that Buyukcekmece water has the highest membrane fouling for PAC/UF system as was the case with PAC/MF system. On the other hand, Terkos water showed the lowest level fouling trends with maximum TMP value of 33 mbar (Figure 9). As shown in Figure 9, Omerli water does not follow the same fouling trend as that for Terkos water, indicating that the extent of fouling might be quite different for the similar percent of hydrophilic content (Terkos: 53%, and Omerli: 50%) adsorbed within PAC cake layer on UF membrane. These results may be attributed to the shape of the organic molecules with source waters. Some of the hydrophilic compounds have linear shape while others have globular shape molecule. Thus, the shape of the molecules affects the fouling trends of membranes. On the other hand, Buyukcekmece water which contained moderate hydrophilic NOM (47%) appeared to exert higher TOC and UV_254_ rejection with PAC/UF system despite its higher fouling quantity. This implies that the degree of membrane fouling in this particular case does not necessarily depend to the extent of TOC removal and apparently this observation distinctively described that the TOC fractions which are more effectively removed by the PAC/UF membrane are not the same fractions that contribute to membrane fouling [8, 43].

## 4. Conclusions 

The conclusions that can be drawn from the results of this investigation are as follows.

- Filtration of Istanbul source waters by a bare MF and UF membranes removed negligible TOC (<20% for MF and 5% for UF); by contrast, significant amounts of TOC (>80% for MF and >65% for UF) were removed when 25 g/L-day PAC were predeposited on the both of membrane surfaces. Since the MF membrane pore diameter is too large compared to molecular size of NOM present in three source waters, the dominant removal mechanism of PAC/MF hybrid system is adsorption of organic matter within PAC layer rather than size exclusion/steric hindrance of NOM by membrane pores.
- Inconsistent with the literature findings, the PAC removed UV_254_ less efficiently than TOC, suggesting that they selectively bind more hydrophilic molecules. Furthermore, these findings indicate that PAC adsorbent preferentially removes aliphatic organics to which UV_254_ is not attributed to.
- The results of this study revealed that higher PAC dose did not reduce the TMP development; on the contrary it prompts the membrane fouling more severe than when no adsorbent was used, even though the PAC adsorbed a significant fraction of the influent NOM. One interpretation of these results is that the PAC particles form gel layers at the membrane surface and that, when the membrane system is pressurized, the gels become compressed and distorted and thereby block the pores on the membrane surface. In addition, it was assumed that the cake layer formed by the PAC particles on the surface of UF membrane is more permeable and thinner than those of MF membrane. This could be explained by the fact that the hydrophilic NOM was not effectively retained by the pore size exclusion and electrostatic repulsion mechanisms as it was relatively smaller in molecular weight than the membrane pore size, possessing greater surface area, and has less electron-rich sites than that of the hydrophobic NOM. This phenomenon enhanced further mass accumulation (adsorption) of the hydrophilic NOM onto the pores that caused significant irreversible fouling by constricting and reducing the effective membrane permeability.
- It is important to note that the presence of dissolved Mg^+2^ and Ca^+2^ in the waters of Buyukcekmece, Terkos, and Omerli has a strong influence on the UF membrane performance. The Ca^+2^ ions can bind with the acidic functional groups of the NOM, elevating the degree of hydrophobicity of the NOM molecules and developing a dense thick fouling layer on the UF membrane surface.
- It was concluded that since all three source waters have similar HPI content, it appears that the PAC interaction with membrane pores was responsible for offsetting the NOM fractional effects on membrane fouling for all three source waters. Besides, hydrophobic/hydrophilic character or the molecular size distribution ratio of NOM plays a quite limited role on PAC cake formation, while a more relevant effect is due to the combination of ionic strength, colloidal movement and PAC pore blockage mechanisms.

## Figures and Tables

**Table 1:** Characterization of surface water source quality parameters [22].

Parameters	Unit	Buyukcekmece	Terkos	Omerli
pH	—	8.20	7.92	7.40
Turbidity	NTU	18	2.39	1.52
Color	mg/L Pt-Co	28	20	10
Conductivity	*μ*S/cm	540	344	278
Alkalinity	mg/L CaCO_3_	139	124	113
Hardness	mg/L CaCO_3_	182	130	114
Ca^+2^	mg/L	161	46	40
TDS	mg/L	258	169	136
Bromide (Br^−^)	*μ*g/L	230	110	50
TOC	mg/L	6.45	6.54	4.52
DOC	mg/L	5.12	5.70	3.75
UV_254_	1/cm	0.144	0.150	0.100
SUVA_254_	L/mg × m	2.81	2.63	2.67

**Table 2:** Typical characteristics of membranes used in this study.

Parameters	MF	UF
Flux rate	150 L/m^2^-h	18–72 L/m^2^-h
Max. operating temperature	40°C	40°C
Max. operating pressure	5.5–3.5 bar	0.60 bar
pH range	5–10	5–9
Effective membrane surface area	—	0.93 m^2^
Membrane material	Polypropylene	Polypropylene
Molecular weight cut-off	0.1 *μ*m	0.04 *μ*m
Membrane type	Hydrophilic	Hydrophilic
